# Bortezomib Induced Hepatitis B Reactivation

**DOI:** 10.1155/2014/964082

**Published:** 2014-05-04

**Authors:** Salwa Hussain, Ruby Jhaj, Samira Ahsan, Muhammad Ahsan, Robert E. Bloom, Syed-Mohammed R. Jafri

**Affiliations:** ^1^Department of Internal Medicine, Providence Hospital and Medical Centers, 16001 W 9 Mile Road, Southfield, MI 48075, USA; ^2^Division of Nephrology, Department of Medicine, Providence Hospital and Medical Centers, 16001 W 9 Mile Road, Southfield, MI 48075, USA; ^3^Division of Hematology & Oncology, Department of Medicine, Providence Hospital and Medical Centers, 16001 W 9 Mile Road, Southfield, MI 48075, USA; ^4^Division of Gastroenterology, Department of Medicine, Henry Ford Health System, Detroit, MI 48202, USA

## Abstract

*Background*. It has recently been reported that hepatitis B (HBV) reactivation often occurs after the use of rituximab and stem cell transplantation in patients with lymphoma who are hepatitis B surface antigen (HBsAg) negative. However, clinical data on HBV reactivation in multiple myeloma (MM) is limited to only a few reported cases. Bortezomib and lenalidomide have remarkable activity in MM with manageable toxicity profiles, but reactivation of viral infections may emerge as a problem. We present a case of MM that developed HBV reactivation after bortezomib and lenalidomide therapy. *Case Report*. A 73-year-old female with a history of marginal cell lymphoma was monitored without requiring therapy. In 2009, she developed MM, presenting as a plasmacytoma requiring vertebral decompression and focal radiation. While receiving radiation she developed renal failure and was started on bortezomib and liposomal doxorubicin. After a transient response to 5 cycles, treatment was switched to lenalidomide. Preceding therapy initiation, her serology indicated resolved infection. Serial monitoring for HBV displayed seroconversion one month after change in therapy. *Conclusion*. Bortezomib associated late HBV reactivation appears to be a unique event that requires further confirmation and brings to discussion whether hepatitis B core positive individuals would benefit from monitoring of HBV activation while on therapy.

## 1. Background

An estimated 800,000–1.4 million people in the United States suffer from chronic hepatitis B infection. Worldwide chronic infection is an even greater problem affecting approximately 350 million individuals [1]. Reactivation of hepatitis B (HBV) refers to the abrupt increase in hepatitis B virus replication in a patient with inactive or resolved HBV. It has been linked to an offense that offsets the host's immunity and permits viral activity. This can be induced by exposure to immunosuppressants or host immunodeficiency, chemotherapy with rituximab, immune modulation using prednisone, or infliximab for autoimmune conditions. Reactivation can also occur with progression of human immunodeficiency virus (HIV) infection or after solid organ transplantation (heart, lung, and kidney). The same process of reactivation is seen after bone marrow and liver transplantation [2, 3].

Two agents commonly used in the treatment of MM include bortezomib (BOR) and lenalidomide (LEN). BOR reversibly inhibits proteasomes and disrupts cell-signaling pathways. LEN has immunomodulatory, antiangiogenic, and apoptotic properties [4]. Both of these agents have remarkable activity in multiple myeloma (MM) with manageable toxicity profiles, but reactivation of viral infections can emerge as a problem [5].

Literature is scarce on HBV reactivation induced by immunosuppressive chemotherapy in MM patients. Here, we present a MM patient who developed HBV reactivation after BOR and LEN therapy.

## 2. Case Report

A 73-year-old Russian female who had history of marginal cell lymphoma diagnosed in 2006 was being monitored every 3 months without requiring any therapy. Other medical comorbidities included diabetes mellitus, hypertension, chronic stable pancytopenia, and a history of resolved HBV infection. The patient did not engage in tobacco, alcohol, or illicit drug use.

In 2008, she presented with back pain and was diagnosed with MM, presenting as a plasmacytoma. This was positive for CD 138 and kappa light chain and negative for LCACD 20 and lambda light chain. She required vertebral decompression and focal radiation. At this time her laboratory findings were as follows: white blood cell count (WBC), 3500/*μ*L; hemoglobin (Hgb), 12.5 g/dL; platelets, 140,000/*μ*L; and creatinine (Cr) 1.0 mg/dL. She developed MM related acute renal failure with Cr of 6.2 mg/dL, which did not improve with plasmapheresis and resulted in dialysis dependence. She was started on BOR and liposomal doxorubicin. BOR was administered at a dose of 1.3 mg/m^2^/day on days 1, 4, 8, and 11 of three weekly cycles. Liposomal doxorubicin at 30 mg/m^2^ and zoledronic acid 4 mg were given on day 1 of each cycle along with ondansetron 16 mg. After a temporary response to five cycles, she progressed and was switched to LEN 5 mg taken after dialysis, thrice a week, on a 3-week-on and one-week-off regimen. She was being monitored for hepatitis serology at the dialysis center and had undergone seroconversion eight months after receiving BOR and one month after initiation of LEN therapy.

Prior to BOR therapy, the patient was seropositive for hepatitis B surface antibody (anti-HBs) and hepatitis B core antibody (anti-HBc) with negative hepatitis B surface antigen (HBsAg), which was indicative of her having a resolved infection. Hepatitis C antibody was negative. Other laboratory values were as follows: WBC, 3000/*μ*L; Hgb, 9.5 g/dL; platelets, 96,000/*μ*L with normal electrolyte values. Her aminotransferase levels at that time were within normal limits [alanine aminotransferase level (ALT) 14 U/L and aspartate aminotransferase level (AST) 22 U/L]. After seroconversion, HBsAg was highly reactive with HBV DNA of 1.2 × 10^6^ IU/mL (6.9 × 10^6^ copies/mL); ALT was 19 U/L, AST 29 U/L, WBC 1900/*μ*L, Hgb 10.8 g/dL, and platelets 142,000/*μ*L. Ultrasound of the abdomen revealed an enlarged liver with coarse echo texture, which was deemed to be secondary to early cirrhosis or hepatitis. Her Naranjo adverse drug reaction probability score was 5 indicating a probable adverse drug reaction [10]. The patient was started on tenofovir 300 mg once a week (dose adjusted for renal impairment). During the following months, she developed cancer related complications. She was admitted to the hospital with vertebral fractures requiring surgical intervention, venous thrombosis, progressive functional decline, and sepsis. She was eventually placed under comfort care and died eleven months after seroconversion.

## 3. Discussion

Reactivation of HBV replication with increase in serum HBV DNA and ALT level has been reported in 20% to 50% of untreated HBV carriers undergoing immunosuppressive or cancer chemotherapy. In most instances, the hepatitis flares are asymptomatic, but icteric flares, and even hepatic decompensation and death have been observed [11, 12]. Clinical studies including two controlled trials showed that prophylactic therapy with antiviral suppression can reduce the rate of HBV reactivation, severity of associated hepatitis flares, and mortality [11, 13]. These studies also demonstrated the ineffectiveness of starting therapy once reactivation had already occurred.

Another randomized, controlled trial of entecavir prophylaxis for rituximab associated HBV reactivation confirmed the reactivation incidence rate of individuals who did not receive prophylaxis as being 17.9%. This was within the range of previous reports and highlighted that entecavir prophylaxis did result in a lower reactivation rate [14]. Interestingly, BOR has been known to significantly increase the risk of reactivation of varicella zoster virus and prophylactic use of acyclovir is recommended. We present a comparison of reported cases of HBV reactivation in Table 1.

Beysel et al. [8] described the case of a 58-year-old male initially in the replicative phase of chronic HBV. He received four courses of VAD (vincristine, doxorubicin, and dexamethasone) regimen while on lamivudine prophylaxis. HBV DNA became undetectable from an initial 350,000 copies/mL (70,000 IU/mL). He was subsequently started on BOR for refractory MM with persisting undetectable HBV DNA levels after nine months of therapy. However, 18 months later, BOR associated late HBV reactivation was demonstrated by detection of 20,000 copies/mL (4000 IU/mL) of HBV DNA. The patient was lost to follow-up and had not taken the recommended lamivudine for at least a nine-month period. This raises the possibility of reactivation secondary to lack of compliance with recommended prophylaxis.

Tanaka et al. [7] reported the case of a 72-year-old male with remote HBV infection who underwent reactivation after receiving 10 courses of BOR and dexamethasone (DEX). Both were immediately discontinued and he was started on entecavir. The patient responded favorably with negative HBV DNA after one month of antiviral therapy. BOR and DEXA were resumed thereafter. The serology of this patient preceding BOR therapy, with isolated positive anti-HBs, favors immunization over resolved infection. The lack of risk factors for contracting infection between onset of BOR and detection of HBV DNA demonstrates how this immunity can be lost and active infection recurs in the setting of chemotherapy.

Goldberg et al. [6] describes a 72-year-old male with resolved HBV who underwent reactivation after four months of treatment with BOR. He failed to respond to entecavir (ETV), developed fulminant hepatic failure, and passed away within 4 weeks of onset of the acute episode. Given the isolated positive HBcAb, this case is the closest in terms of the initial serologies of our case, with reactivation of an apparently resolved hepatitis. With BOR and the ensuing immunocompromise, this infection resulted in fulminant hepatic failure. Our patient, on the other hand, had a progressive clinical decline secondary to complications of MM resulting in the family's decision to withdraw care. The role of one month of LEN remains unclear in this reactivation process.

Mya et al. conducted a systematic analysis investigating the prevalence of HBV infection and the incidence of HBV reactivation among MM patients. Of the 273 patients with MM, one had reactivation four months after BOR and DEX salvage therapy. Although data are limited, it has been suggested that BOR may alter the number and function of specific lymphocyte subsets, in particular the CD8 T cells and CD56 natural killer cells [15]. The consequent suppression of cellular mediated immunity (CMI) that plays an important role in the suppression of VZV reactivation may have a role in the unchecked replication of VZV during BOR therapy [16]. As HBV is another DNA virus that remains dormant in the human host, whether HBV reactivation can be attributable to BOR's impact on CMI remains to be defined.

AASLD chronic HBV guidelines 2009 [9] (Table 2) state that HBsAg and anti-HBc testing should be performed in persons who have high risk of HBV infection [17], prior to initiation of chemo- or immunosuppressive therapy. Prophylactic antiviral therapy should be administered to HBV carriers (regardless of baseline serum HBV DNA level) at the onset of cancer chemotherapy or a finite course of immunosuppressive therapy and maintained for 6 months afterwards. Viral relapse after withdrawal of lamivudine has been reported in patients with high prechemotherapy HBV DNA level. HBsAg-positive persons with serum HBV DNA levels >2,000 IU/mL prior to undergoing cytotoxic chemotherapy should continue antiviral therapy until they reach therapeutic endpoints for chronic HBV.

We focus our attention on the current recommendations in reference to serologies as applies to our patient. AASLD guidelines state that while HBV reactivation can occur in persons who are HBsAg negative but anti-HBc and anti-HBs positive and in those with isolated anti-HBc, this is an infrequent phenomenon. There is no enough information to recommend routine prophylaxis for these individuals. These patients should be monitored with serial laboratory testing and antiviral therapy initiated if serum HBV DNA becomes detectable [9].

## 4. Conclusion

In conclusion, it has been a known fact that some chemotherapeutic agents pose a significant risk of HBV reactivation in HBV carriers. Bortezomib associated reactivation in resolved HBV is a unique phenomenon and brings to discussion whether such individuals receiving chemotherapy would benefit from antiviral prophylaxis versus serial monitoring of HBV DNA. This question should be addressed by future research studies.

## Figures and Tables

**Table 1:** Cases reported in the literature on HBV reactivation secondary to bortezomib (BOR) therapy.

Case and year reported [reference]		Serology	Before BOR	After BOR	HBV reactivation time	Outcome
Goldberg et al., 2013 [6]	72-year-old white male with MM failed therapy with thalidomide and LEN, treated with BOR	HBsAg HbsAb HBcAb HBVDNA	Negative Negative Positive UD	Positive Negative NA 1.7 × 10^8^ IU/mL	4 months after BOR	Fatal fulminant hepatic failure

Tanaka et al., 2012 [7]	72-year-old Japanese male with MM resistant to melphalan, prednisolone, and thalidomide, treated with BOR	HBsAg HbsAb HBcAb HBVDNA	Negative Positive Negative UD	NR NR NA 2.7 × 10^5^ copies/mL	10 cycles after BOR	HBV resolved after 4 weeks of entecavir with BOR resumed thereafter

Beysal et al., 2009 [8]	58-year-old male with light chain MM treated with combination chemo followed by BOR	HBsAg HbeAg HBcAb HBVDNA	Positive Positive NA Initially 350,000 copies/mL which became UD with lamivudine	NR NR NA 20,000 copies/mL	18 months after BOR	Unclear postreactivation course

BOR: bortezomib, HBsAg: hepatitis B surface antigen, HbsAb: hepatitis B surface antibody, HBcAb: hepatitis B core antibody, HbeAg: hepatitis B e antigen, HBVDNA: hepatitis B virus deoxyribonucleic acid, NA: not applicable, and UD: undetectable.

**Table 2:** AASLD guidelines: recommendations for treatment of hepatitis B carriers who require immunosuppressive or cytotoxic therapy [9].

AASLD guidelines 2009	Strength of recommendation
HBsAg and anti-HBc testing should be performed in patients who are at high risk of HBV infection, prior to initiation of chemotherapy or immunosuppressive therapy.	II-3
Prophylactic antiviral therapy is recommended for HBV carriers at the onset of cancer chemotherapy or of a finite course of immunosuppressive therapy.	
(a) Patients with baseline HBV DNA<2,000 IU/mL level should continue treatment for 6 months after completion of chemotherapy or immunosuppressive therapy.	III
(b) Patients with high baseline HBV DNA (>2,000 IU/mL) level should continue treatment until they reach treatment endpoints as in immunocompetent patients.	III
(c) Lamivudine or telbivudine can be used if the anticipated duration of treatment is short (<12 months) and baseline serum HBV DNA is not detectable.	(I for lamivudine and III for telbivudine)
(d) Tenofovir or entecavir is preferred if longer duration of treatment is anticipated.	III
(e) IFN*α* should be avoided in view of the bone marrow suppressive effect.	II-3

Quality of evidence on which a recommendation is based: I: randomized controlled trials, II-1: controlled trials without randomization, II-2: cohort or case-control analytic studies, II-3: multiple time series, dramatic uncontrolled experiments, and III: opinions of respected authorities and descriptive epidemiology.
